# A case-control analysis: changes in gut microbiota composition in children with autism spectrum disorder

**DOI:** 10.3389/fmicb.2026.1890167

**Published:** 2026-08-25

**Authors:** Linlin Fan, Ying Tian, Jiayi Jin, Na Zhang, Caihong Sun, Yang Liu, Le Kang

**Affiliations:** 1 Department of Child Health Care, Yantai Affiliated Hospital of Shandong Medical and Pharmaceutical University, Yantai, China; 2 Department of Neonatology, Yantai Affiliated Hospital of Shandong Medical and Pharmaceutical University, Yantai, China; 3 School of Pharmacy, Shandong Medical and Pharmaceutical University, Yantai, China; 4 Department of Gynecology, Jiaoqiao Central Health Center, Jiaoqiao, China; 5 School of Special Education and Rehabilitation, Shandong Medical and Pharmaceutical University, Yantai, China; 6 Department of Hand Surgery & Microsurgery and Foot & Ankle Surgery, Yantai Affiliated Hospital of Shandong Medical and Pharmaceutical University, Yantai, China

**Keywords:** 16S rRNA sequencing, autism spectrum disorder, case-control study, gut microbiota, microbial dysbiosis

## Abstract

**Background:**

Gut microbiota dysbiosis has been increasingly implicated in autism spectrum disorder (ASD), with the gut-brain axis proposed as a potential mechanistic link. However, ASD-specific microbial signatures remain inconsistent across studies, and whether reported associations reflect primary dysbiosis or ASD-related confounders such as dietary restriction and gastrointestinal comorbidities remains debated.

**Methods:**

A case-control study was conducted enrolling 54 children with ASD and 46 age- and sex-matched typically developing (TD) children. Full-length 16S rRNA gene sequencing was performed on the PacBio Sequel IIe third-generation platform using universal primers 27F/1492R, generating high-fidelity (HiFi) reads via on-instrument circular consensus sequencing (CCS). Gut microbiota differences were assessed using α- and β-diversity analyses, phylum-level composition, Gut Microbiome Health Index (GMHI), Microbial Dysbiosis Index (MDI), differential abundance testing, and LEfSe analysis.

**Results:**

No significant differences were observed in α-diversity between groups, whereas significant differences were identified in β-diversity, GMHI, and MDI. Phylum-level analysis revealed a significantly reduced Bacillota/Bacteroidota ratio in the ASD group. More than 80% of ASD samples had negative GMHI values vs. more than 75% of TD samples with positive values, demonstrating superior discriminative performance compared with traditional diversity indices. Differential abundance analysis identified 15 differentially abundant species: 10 enriched in the TD group, predominantly butyrate-producing bacteria and Bifidobacterium spp., and 5 enriched in the ASD group, predominantly Bacteroides-affiliated taxa. LEfSe confirmed 16 differentially abundant taxa (LDA score ≥ 3.0).

**Conclusion:**

Children with ASD exhibited significant differences in gut microbiota composition compared with TD children, characterized by a functional compositional imbalance-systematic depletion of butyrate-producing bacteria and *Bifidobacterium spp*. alongside enrichment of specific Bacteroides members-rather than alterations in overall species diversity. These findings are consistent with gut-brain axis dysregulation in ASD, though the cross-sectional design precludes causal inference and the possibility that observed differences reflect ASD-related dietary behavior cannot be excluded. GMHI demonstrated superior discriminative performance compared with traditional diversity indices in this cohort, though its application in pediatric ASD populations remains exploratory and requires independent validation in larger multicenter cohorts.

## 1 Introduction

Autism Spectrum Disorder (ASD) is a complex neurodevelopmental disorder characterized by persistent deficits in social communication and social interaction, as well as restricted and repetitive behaviors (Litman et al., 2025). Globally, the reported prevalence of ASD ranges from 0.01% to 4.36%, with a median of 1% (Zeidan et al., 2022). The most recently released official data from the United States indicate that the overall prevalence of ASD has reached 2.76% (1 in 36 children), which is significantly higher than estimates from 2000 to 2018, with a continuous upward trend (Christensen et al., 2016). The medical care and support needs of children with ASD have emerged as a critical public health concern, imposing substantial burdens on families and society. Accordingly, numerous researchers worldwide have devoted themselves to this field of investigation. The etiology of ASD is multifactorial, involving complex interactions between genetic susceptibility and environmental factors (Wang et al., 2025). Although extensive research has been conducted globally, the underlying pathophysiological mechanisms remain incompletely elucidated, and effective interventions are still limited. Notably, numerous clinical observations have revealed that children with ASD frequently present with a high incidence of gastrointestinal comorbidities, such as constipation, diarrhea, and abdominal pain, which are generally more severe than those observed in typically developing children (Alqoaer et al., 2024). These findings have prompted researchers to further investigate the potential role of gut dysfunction in the pathogenesis of ASD.

In recent years, with the rapid development of microbiome research, the concept of the “gut-brain axis” has attracted considerable attention (Goralczyk-Binkowska et al., 2022). As the largest microbial ecosystem in the human body, the gut microbiota communicates bidirectionally with the central nervous system through multiple pathways, including immune signaling, neuroendocrine signaling, the vagus nerve, and metabolic pathways (Dong and Mayer, 2024; Khan et al., 2025). The gut microbiota may influence neurodevelopment through several mechanisms. First, gut microbes produce various neuroactive compounds, including neurotransmitters (e.g., γ-aminobutyric acid, serotonin, and dopamine) and their precursors, which can modulate brain function and behavior (Dicks, 2025). Importantly, approximately 90% of the body's serotonin is produced in the gut, highlighting the potential influence of the gut microbiota on mood and behavioral regulation (Kwon et al., 2019). Second, microbial metabolites, particularly short-chain fatty acids (SCFAs) such as butyrate, propionate, and acetate, are generated by the gut microbiota through fermentation of dietary fiber. These metabolites can enter the systemic circulation and partially cross the blood-brain barrier, thereby exerting neuroprotective effects (Shen et al., 2024; Trompette et al., 2022; Xiao et al., 2022). Third, the gut microbiota regulates the development and function of the immune system. Dysbiosis can induce chronic low-grade inflammation and immune imbalance, and such persistent inflammatory status is closely associated with the onset and exacerbation of various neurodevelopmental disorders, including ASD (Palanivelu et al., 2024; Chen et al., 2025). Fourth, alterations in gut microbiota composition may impair intestinal barrier integrity, leading to increased permeability, translocation of microbial components, and subsequent activation of systemic inflammatory responses (Liu et al., 2025; Gámez-Macías et al., 2024).

Using 16S rRNA gene sequencing, several studies have reported differences in gut microbiota diversity and composition between children with ASD and typically developing (TD) children (Zeidan et al., 2022; Kang et al., 2013). However, findings across different studies have been inconsistent and even contradictory. For instance, some studies have reported decreased α-diversity in individuals with ASD, while others reported no difference or even an increase (Dan et al., 2020; Ha et al., 2021). Heterogeneous results also exist regarding alterations in specific genera, such as *Clostridium, Bifidobacterium*, and *Prevotella* (Zeidan et al., 2022; Dan et al., 2020). Such inconsistencies may be attributed to differences in ethnicity, age, geographical distribution, dietary habits, sample size, and methodological factors (e.g., sequencing platform, gene coverage, and bioinformatic pipelines) across studies.

However, not all studies have reported consistent or significant alterations in the gut microbiota of individuals with ASD. Several investigations have identified limited or non-significant differences in gut microbial composition between ASD and neurotypical individuals, or have argued that previously reported associations may largely reflect the confounding influence of variables such as dietary habits, medication use, and gastrointestinal comorbidities, rather than ASD-specific dysbiosis (Mitchell et al., 2026; Yap et al., 2021; Xiao et al., 2025; Korteniemi et al., 2023). These contradictory findings underscore the importance of rigorous confounding factor control and the need for cautious interpretation of microbiome studies in ASD.

Despite these advances, notable limitations remain in this field. Most existing evidence is based on Western populations, whereas data from Asian children (especially Chinese children) are still scarce. In addition, many studies are limited by small sample sizes or fail to adequately control for potential confounding factors including diet, medication, and gastrointestinal comorbidities. Therefore, we group conducted a well-designed case-control study in the eastern coastal region of China (Yantai, Shandong Province) to systematically characterize the alterations of gut microbiota in children with ASD. This study holds significant value for elucidating the role of the gut microbiota in ASD and identifying population- or region-specific microbial biomarkers.

## 2 Materials and methods

### 2.1 Study Subjects and Ethical Statement

This study was designed as a case-control study. The study protocol was approved by the Ethics Review Committee of Yantai Affiliated Hospital of Shandong Medical and Pharmaceutical University (Approval No.: 20230910100). Written informed consent was obtained from the legal guardians of all participants after full explanation of the study protocol.

#### 2.1.1 ASD group

From October 2023 to November 2023, 54 children with ASD who met the diagnostic criteria were recruited from the outpatient clinics of Yantai Affiliated Hospital of Shandong Medical and Pharmaceutical University. Inclusion criteria: (1) Aged 3-13 years; (2) Diagnosed with autism spectrum disorder independently and concordantly by at least two child psychiatrists or developmental-behavioral pediatricians holding the title of associate chief physician or above, according to the *Diagnostic and Statistical Manual of Mental Disorders, Fifth Edition* (DSM-5); (3) Met the diagnostic cutoff score of the Autism Diagnostic Observation Schedule, Second Edition (ADOS-2), and exhibited significant autistic behavioral characteristics confirmed by the Autism Behavior Checklist (ABC) and/or Childhood Autism Rating Scale (CARS); (4) Able to cooperate with fecal sample collection.Exclusion criteria: (a) Acute gastroenteritis within the past month, or use of antibiotics, prebiotics, probiotics, proton pump inhibitors, or other microecological agents within the past month; (b) Confirmed gastrointestinal diseases (e.g., inflammatory bowel disease, celiac disease), or intestinal surgery within the past 3 months; (c) Severe immune system diseases, congenital metabolic diseases, endocrine disorders (e.g., diabetes, thyroid dysfunction), or chronic infections; (d) Severe neurological diseases (e.g., epilepsy, cerebral palsy).

#### 2.1.2 TD group

During the same period, 46 typically developing children with age- and sex-matched to the ASD group were recruited through community recruitment and kindergarten physical examinations.Inclusion criteria: (1) Aged 3–13 years; (2) Developmental quotient (DQ) within the normal range (generally ≥ 85) assessed by the Denver Developmental Screening Test (DDST); (3) Screening score on the Autism Behavior Checklist (ABC) was far below the clinical cutoff; (4) No history of neuropsychiatric disorders and normal growth and developmental trajectory; (5) No family history of ASD: no confirmed or suspected diagnosis of ASD in first-degree relatives (siblings and parents). Exclusion criteria were identical to those applied to the ASD group. Baseline demographic information (age, sex, mode of delivery, feeding history, etc.), gastrointestinal symptoms (assessed using the 6-item Gastrointestinal Severity Index [6-GSI]), and dietary preferences were collected from all enrolled children via standardized questionnaire.

### 2.2 Fecal Sample Collection and DNA Extraction

Sterile fecal collection kits containing a sample preservation solution were provided to the guardians of all participants. Fresh fecal samples were collected via rectal swabs. All subjects were required to fast for 12 h before sampling. Before collection, the perianal region was thoroughly cleaned with soap, water, and 75% ethanol. A sterile swab was inserted 4–5 cm into the rectum and rotated gently to obtain the fecal material. Samples were immediately stored in sterile tubes at −80 °C in an ultra-low temperature freezer until DNA extraction. Total microbial genomic DNA was extracted from fecal samples of ASD and TD children using the FastPure Stool DNA Isolation Kit (MJYH, Shanghai, China) according to the manufacturer's instructions. DNA integrity was assessed by 1% agarose gel electrophoresis, and DNA concentration, purity, and quality were determined using a NanoDrop 2000 spectrophotometer (Thermo Scientific, USA). DNA samples meeting quality criteria were stored at −20 °C until further use.

### 2.3 Amplification of 16S rRNA gene and high-throughput sequencing

Extracted DNA was used as template for PCR amplification of the full-length 16S rRNA gene or full-length ITS region using barcoded primers 27F (5′-AGRGTTYGATYMTGGCTCAG-3′) and 1492R (5′ -RGYTACCTTGTTACGACTT-3′) (Liu et al., 2016), or ITS1F (5′ -CTTGGTCATTTAGAGGAAGTAA-3′) and ITS4R (5′ -TCCTCCGCTTATTGATATGC-3′) (Chen et al., 2018), respectively. Each 20 μL PCR reaction contained 4 μL 5 × FastPfu buffer, 2 μL 2.5 mM dNTPs, 0.8 μL each of forward and reverse primer (5 μM), 0.4 μL FastPfu polymerase, 0.2 μL BSA, and 10 ng template DNA. Amplification was performed in triplicate under the following conditions: initial denaturation at 95 °C for 3 min, followed by 27 cycles of 95 °C for 30 s, 60 °C for 30 s, and 72 °C for 30 s, with a final extension at 72 °C for 10 min. PCR products were verified by 2% agarose gel electrophoresis, purified using magnetic beads, and quantified with a Qubit 4.0 fluorometer (Thermo Fisher Scientific, USA). Purified amplicons were pooled in proportions corresponding to the required sequencing depth per sample. Libraries were constructed using the SMRTbell Prep Kit 3.0 through sequential steps of DNA damage repair, end repair, and adapter ligation, and sequenced on a PacBio Sequel IIe system (Meiji Biomedicine Technology Co., Ltd., Shanghai, China). HiFi reads were generated from sequencing subreads using the CCS mode in SMRT-Link v11.0 for downstream data analysis.

### 2.4 Bioinformatic analysis

Raw sequencing data were demultiplexed by barcode sequences, followed by length filtering and orientation correction; sequences of 1,000–1,800 bp (16S rRNA) or 300–900 bp (ITS) were retained for downstream analysis. OTU clustering was performed at 97% sequence similarity and chimeric sequences were removed using UPARSE v7.1 (Magoč and Salzberg, 2011) (http://drive5.com/uparse/). To minimize the effect of uneven sequencing depth on alpha and beta diversity analyses, all samples were rarefied to 6,000 sequences; following rarefaction, the mean Good's coverage across samples remained 99.09%, indicating adequate sequencing depth. Taxonomic classification of OTUs was performed using the RDP Classifier v2.11 (http://rdp.cme.msu.edu/) against the SILVA 16S rRNA gene database (v138) at a confidence threshold of 70%, and community composition was summarized at multiple taxonomic levels. Alpha diversity was assessed using four indices: ACE and Chao indices reflect species richness (higher values indicate greater richness), while Simpson and Shannon indices reflect community diversity (lower Simpson or higher Shannon values indicate higher diversity). Functional prediction of 16S rRNA data was conducted using PICRUSt2 v2.2.0 (Wang et al., 2007). All bioinformatic analyses were performed on the Majorbio Cloud Platform (https://cloud.majorbio.com).

## 3 Results

### 3.1 Baseline characteristics of subjects, sequencing quality, and general profile of gut microbiota

A total of 54 children with ASD and 46 TD children were enrolled in this study. The clinical characteristics of the children with ASD are summarized in Table 1. The mean ABC score was 81.2 ± 14.6 (range: 68–125), and all children met the diagnostic criteria for moderate to severe ASD. Gastrointestinal symptoms were highly prevalent in the ASD group. Overall, 75.9% of children with ASD presented at least one gastrointestinal symptom, among which constipation was the most common (55.6%), followed by diarrhea (27.8%) and abdominal pain (22.2%). No severe gastrointestinal symptoms such as mucous stool or hematochezia were observed. Furthermore, 48.1% of children with ASD exhibited marked picky eating behavior and food selectivity.

**Table 1:** Clinical characteristics of children with ASD (*n* = 54).

Characteristics	Value/*n* (%)
15.6-7.2,-1.3242pt ABC score	81.2 ± 14.6 (68-125)
Gastrointestinal symptoms	
Constipation	30 (55.6%)
Abdominal pain	12 (22.2%)
Diarrhea	15 (27.8%)
15.6-7.2,-1.3242ptAt least one GI symptom	41 (75.9%)
Dietary behavior	
Food selectivity/Picky eating	26 (48.1%)

Data are presented as mean ± standard deviation or n (%). ABC, Autism Behavior Checklist; ASD, autism spectrum disorder; GI, gastrointestinal.

**Figure 1:**
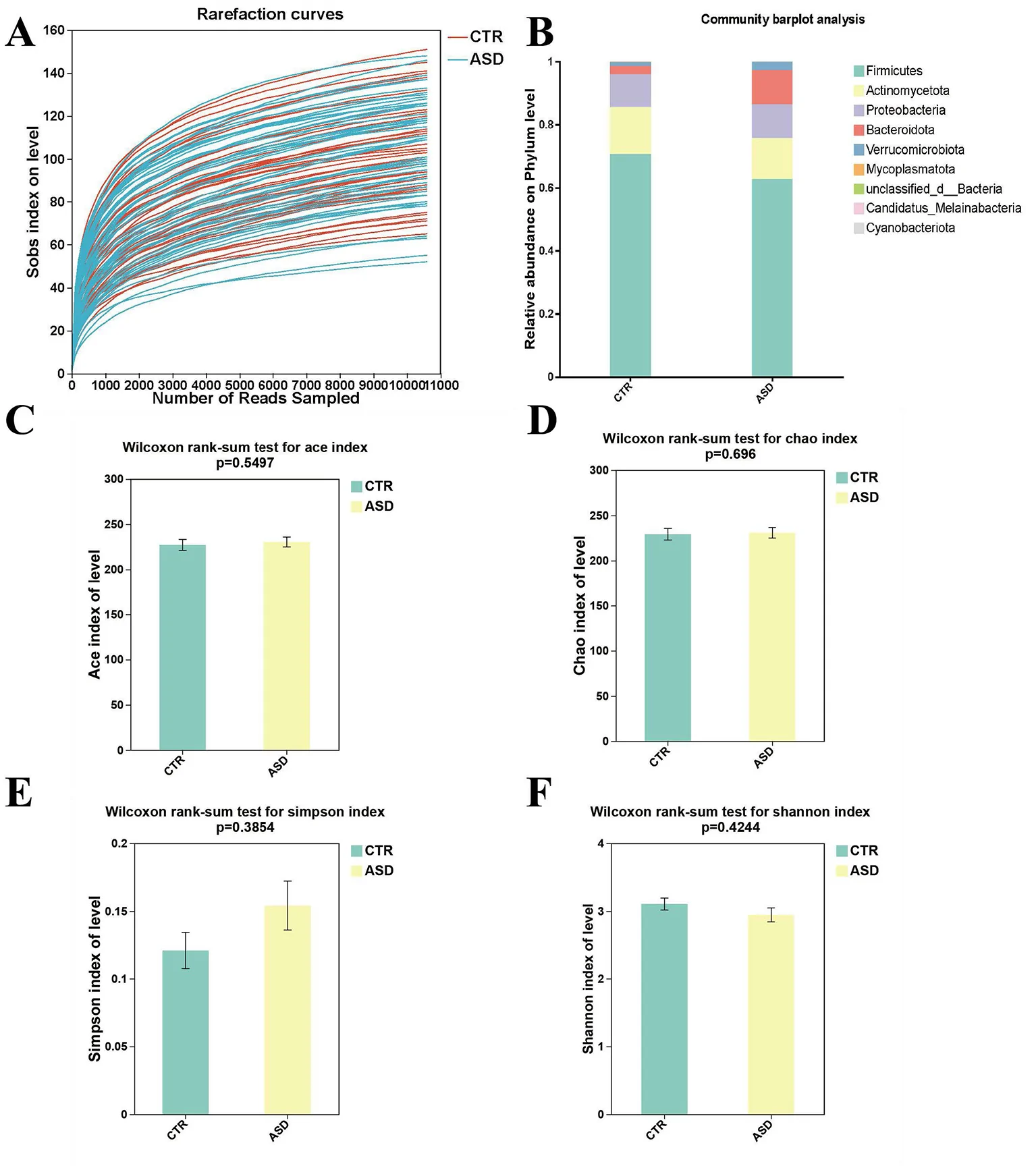
Phylum-level composition and diversity comparison of gut microbiota between two groups. **(A)** Rarefaction curves. **(B)** Community barplot at phylum level. **(C)** ACE index comparison. **(D)** Chao index comparison. **(E)** Simpson index comparison. **(F)** Shannon index comparison.

A total of 100 fecal samples were subjected to 16S rRNA gene sequencing. After quality control and filtering, 3,398,061 high-quality sequences were obtained, with a total base number of 4,932,055,418 bp, an average sequence length of 1,451 bp, and an average of 33,981 sequences per sample. Rarefaction curves reached saturation for all samples (Figure 1A).

Taxonomic annotation was performed against the SILVA 16S rRNA gene database (v138) using the RDP Classifier (v2.11) with a confidence threshold of 70%. All high-quality sequences were successfully classified at different taxonomic levels. Specifically, all sequences belonged to 1 domain (Bacteria) and 1 kingdom (Bacteria), and were further assigned to 11 phyla, 16 classes, 41 orders, 76 families, 191 genera, and 404 species. Clustering analysis was conducted at a 97% sequence similarity threshold, and 573 operational taxonomic units (OTUs) were identified.

To characterize compositional differences in gut microbiota between ASD and TD children, we first analyzed the community structure at the phylum level. As shown in the community bar plot (Figure 1B), Bacillota was the absolutely dominant phylum in both groups. In the CTR group, the average relative abundance of Bacillota was as high as 70.83%, followed by Actinomycetota (14.85%) and Proteobacteria (10.33%), while Bacteroidota was relatively low (2.67%). These four dominant phyla accounted for 98.68% of the total microbiota, forming the core composition of gut microbiota in healthy children.

To evaluate species richness and community diversity of gut microbiota in children with ASD, four α-diversity indices were calculated (Figures 1C–F). In terms of species richness, the ACE and Chao indices were higher in the ASD group than in the CTR group, but the between-group differences were not statistically significant (ACE: *P* = 0.5497; Chao: *P* = 0.696). Regarding community diversity, the ASD group showed an opposite trend: the Simpson index was increased, whereas the Shannon index was decreased compared with the CTR group. A higher Simpson index and lower Shannon index jointly suggest a potential tendency toward reduced microbial diversity and simplified community structure in the ASD group; however, these differences did not reach statistical significance under the current sample size.

### 3.2 Analysis of gut microbiota composition and differential species

**Figure 2:**
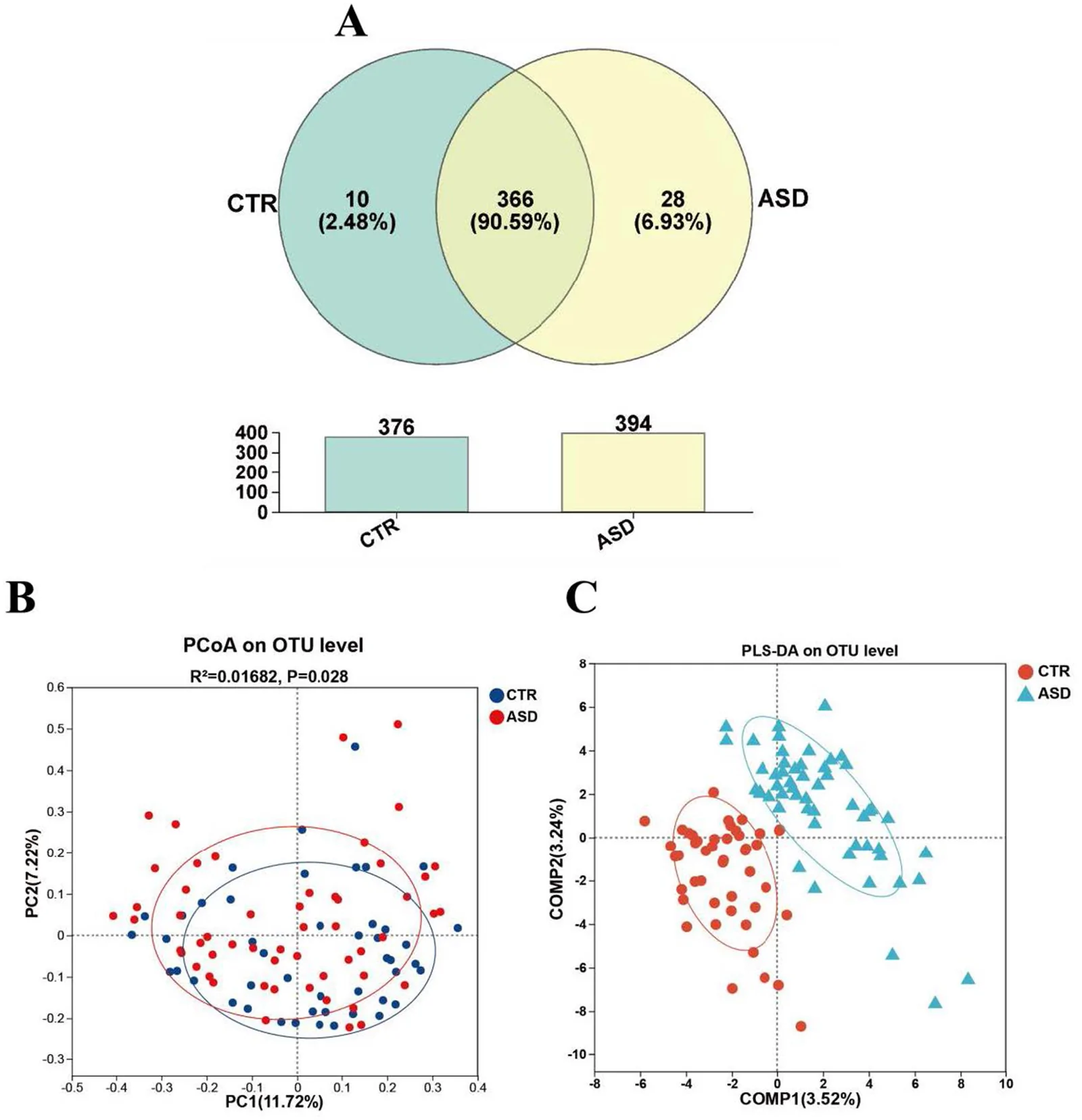
Species composition characteristics and β-diversity analysis of gut microbiota between two groups. **(A)** Venn diagram at species level and species count bar chart. **(B)** Principal coordinates analysis (PCoA) at OTU level. **(C)** Partial least squares discriminant analysis (PLS-DA) at OTU level. (Note: The Venn diagram is presented in classical (non-proportional) format; the actual number of species in each subset is indicated by the numerical labels within each region, and visual area does not represent proportional abundance).

To assess the species-level compositional characteristics of gut microbiota between children with ASD and TD children, Venn diagram analysis was performed (Figure 2A). A total of 376 species were detected in the CTR group and 394 species in the ASD group, among which 366 species were shared by both groups. The shared species accounted for 97.34% of total species in the CTR group and 92.89% in the ASD group. The overall proportion of shared species reached 90.59%, indicating that the two groups exhibited a highly consistent basic framework of species composition, which together constituted the core gut microbiota of children. Although shared species predominated, a certain number of specific species were also observed: 10 unique species in the CTR group and 28 unique species in the ASD group. The number of unique species in the ASD group was 2.8 times that in the CTR group, suggesting that specific microbial colonization patterns or microenvironmental changes may exist in the gut of children with ASD, allowing the colonization of some low-abundance species.

Beta diversity analysis was applied to evaluate differences in microbial community composition among samples. To comprehensively dissect the structural differences in gut microbiota between ASD and TD children, two complementary dimensionality-reduction methods—Principal Coordinates Analysis (PCoA) and Partial Least Squares Discriminant Analysis (PLS-DA)—were used for systematic evaluation. The results showed that samples from the CTR and ASD groups exhibited a clear separation trend in the two-dimensional coordinate space (Figure 2B). The first two principal coordinates, PC1 and PC2, explained 11.72% and 7.22% of the variation in community composition, respectively, with a cumulative contribution rate of 18.94%. This explanatory power was comparable to that reported in previous gut microbiota studies of ASD, consistent with the high-dimensional and complex nature of microbiome data. Statistical testing using Adonis demonstrated significant differences in gut microbial community composition between the two groups (*R*^2^ = 0.01682, *P* = 0.028), confirming that the overall gut microbial structure differed significantly between children with ASD and TD children. It should be noted, however, that the *R*^2^ value indicates that ASD group membership explains approximately 1.68% of the total variance in gut microbial composition, which is consistent with the high inter-individual variability that characterizes gut microbiome data and is comparable to *R*^2^ values reported in other microbiome case-control studies of ASD and related neurodevelopmental conditions.

To further improve the discriminatory power of intergroup differences, supervised PLS-DA was used for validation. In the PLS-DA score plot at the OTU level, samples from the CTR and ASD groups showed an even clearer separation pattern (Figure 2C), with samples from each group clustering in distinct regions and obvious boundaries between groups. It should be noted that PLS-DA is a supervised method and its visual separation pattern may be partially influenced by overfitting. The robustness of the model was assessed by permutation testing (999 permutations), and the results confirmed that the observed discrimination exceeded chance levels. The unsupervised PCoA analysis with Adonis testing (*R*^2^ = 0.01682, *P* = 0.028) provides independent, overfitting-free confirmation of significant intergroup differences in gut microbial community composition. Together, PCoA and PLS-DA confirmed significant differences in overall gut microbial community structure between ASD and TD children.

### 3.3 Analysis of Gut Microbiome Health Index (GMHI) and Microbial Dysbiosis Index (MDI)

**Figure 3:**
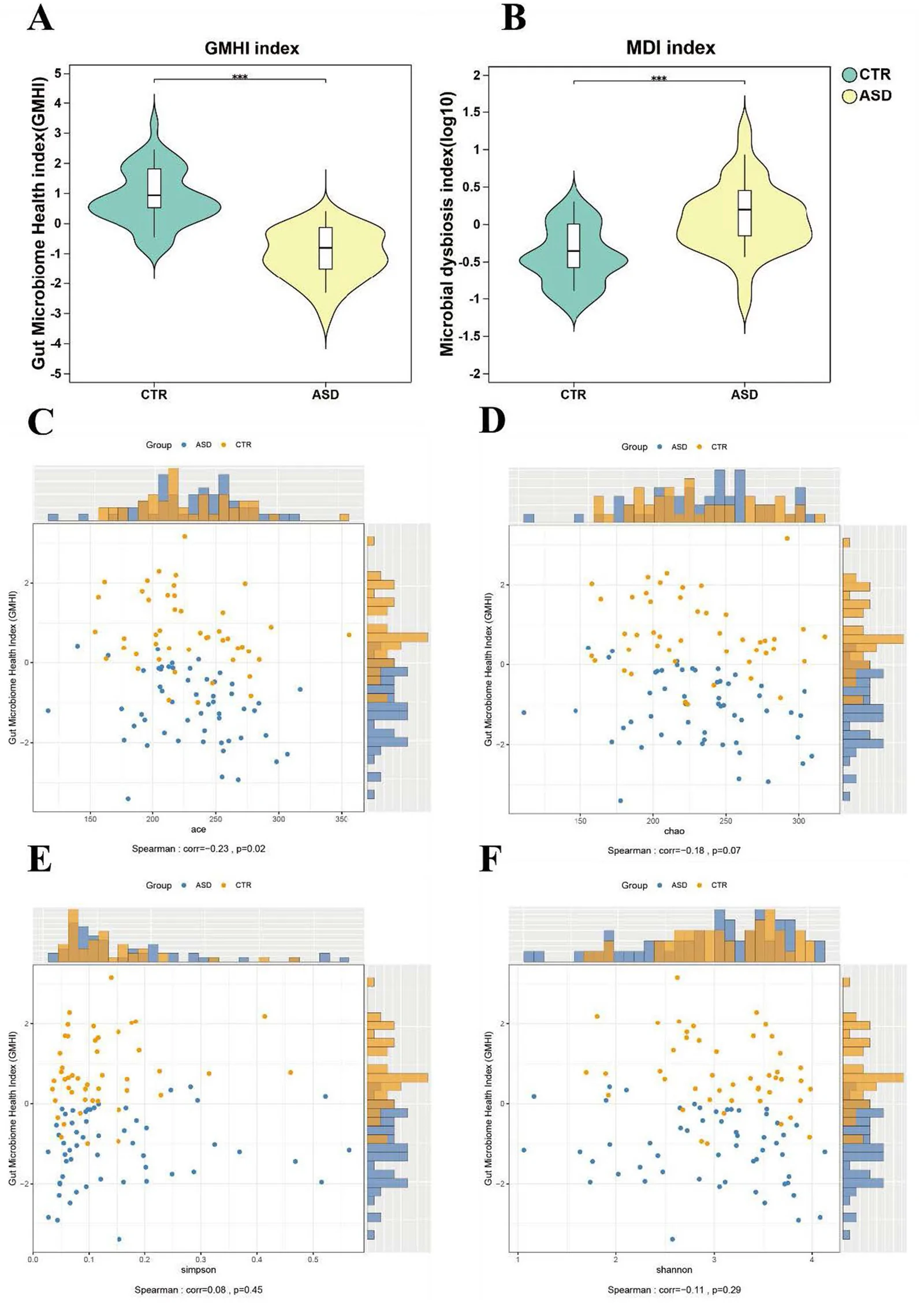
Comparative analysis of gut microbiome health indices and traditional diversity indices. **(A)** Comparison of Gut Microbiome Health Index (GMHI) between groups (***P < 0.001). **(B)** Comparison of Microbial Dysbiosis Index (MDI) between groups (***P < 0.001). **(C)** Correlation analysis between GMHI and ACE index. **(D)** Correlation analysis between GMHI and Chao index. **(E)** Correlation analysis between GMHI and Simpson index. **(F)** Correlation analysis between GMHI and Shannon index.

On the basis of traditional diversity indices, the Gut Microbiome Health Index (GMHI) and Microbial Dysbiosis Index (MDI) were introduced in this study to more comprehensively evaluate the overall gut microbiome health status in children with ASD. Between-group comparison showed that GMHI values differed extremely significantly between the CTR and ASD groups (*P* < 0.001; Figure 3A). Violin plots revealed completely distinct distribution patterns between the two groups: GMHI values in the CTR group were concentrated in the positive range, whereas those in the ASD group were significantly shifted toward negative values. The distributions of the two groups were almost completely separated, with only slight overlap between −0.5 and +1.0. A negative GMHI indicates that the relative abundance of disease-associated bacteria exceeds that of health-associated bacteria. This distribution pattern demonstrates that the overall gut microbiome health of children with ASD is significantly poorer than that of TD children, with obvious characteristics of ecological dysbiosis. These results are highly consistent with the frequent gastrointestinal symptoms (constipation, diarrhea, abdominal pain) observed in children with ASD, further supporting that gut microecological imbalance may be one of the important pathological features of ASD.Similarly, MDI analysis also showed an extremely significant difference between the CTR and ASD groups (*P* < 0.001; Figure 3B). The MDI value in the ASD group was significantly higher than that in the CTR group, further verifying the dysbiotic status of the gut microbiome in children with ASD. The consistent results of the two indices confirmed ASD-related gut microecological disturbance from different perspectives.

To evaluate the performance of GMHI in distinguishing ASD and TD children compared with traditional diversity indices, we systematically compared the discriminatory ability of GMHI with multiple α-diversity indices (Figures 3C–F). Scatter plots combined with marginal histograms clearly illustrated the between-group separation of each index. In the comparison between GMHI and ACE index (Figure 3C), the ACE index (X-axis) showed extensive overlap between the two groups, and the marginal histograms showed severe overlapping distributions, failing to form an effective discriminative boundary. In contrast, GMHI (Y-axis) clearly separated the two groups vertically: most CTR samples were distributed in the positive region (GMHI > 0), while most ASD samples were in the negative region (GMHI < 0). The right marginal histogram exhibited an obvious bimodal distribution, allowing good discrimination between groups using 0 as the cutoff. Spearman correlation analysis showed a weak negative correlation between ACE index and GMHI (*r* = −0.23, *P* = 0.02), suggesting that they reflect gut microbiome characteristics from different dimensions to some extent.

A similar pattern was observed in the comparison between GMHI and Chao index (Figure 3D). The Chao index showed high overlap between groups, whereas GMHI retained good discriminatory power. In the comparison between GMHI and Simpson index (Figure 3E), the Simpson index also showed extensive overlap, with almost completely mixed distributions in the top histogram. GMHI still maintained clear between-group separation, and no significant correlation was observed between them (*r* = 0.08, *P* = 0.45), indicating a weak association between community evenness and gut health status. The comparison between GMHI and Shannon index (Figure 3F) further strengthened the above findings. The Shannon index showed extensive overlap and poor separation between groups. In contrast, the right histogram of GMHI displayed a clear bimodal pattern with distinct boundaries.

Although GMHI cannot be used alone for ASD diagnosis, its potential as a research-grade index for monitoring gut microecological changes in intervention studies warrants further investigation. We emphasize that the clinical applicability of GMHI in pediatric ASD populations should not be inferred from the present findings alone, as independent replication in larger, multicenter, age-stratified pediatric cohorts is a prerequisite before GMHI can be considered a candidate biomarker for clinical application in ASD.

### 3.4 Analysis of differential gut microbiota between children with ASD and TD children

**Figure 4:**
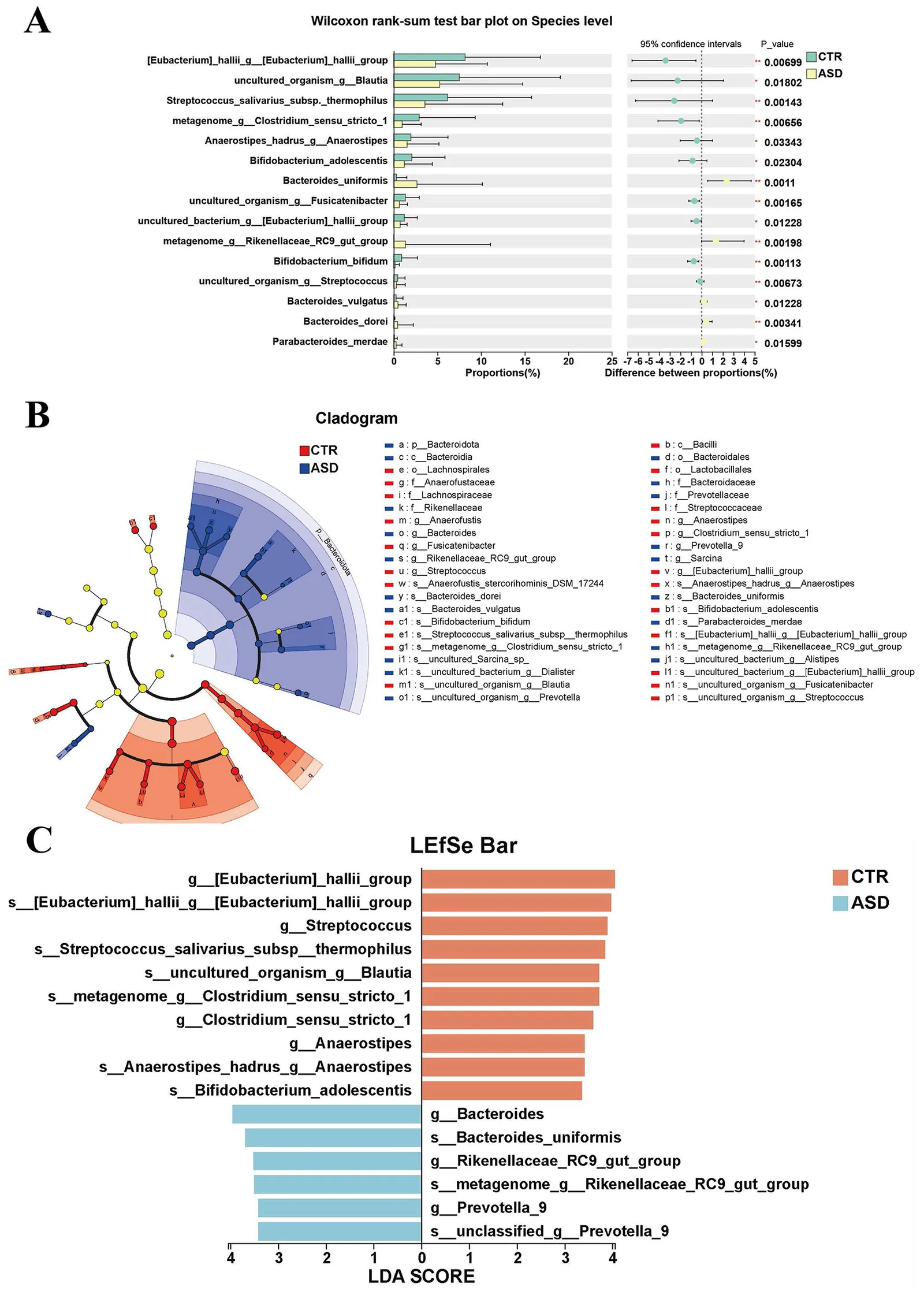
Differential species identification and LEfSe analysis of gut microbiota between two groups. **(A)** Wilcoxon rank-sum test bar plot at species level. Left side shows relative abundance (%) of each species, right side shows difference between proportions (%) with 95% confidence intervals, (*P < 0.05, **P < 0.01). **(B)** LEfSe analysis cladogram showing phylogenetic distribution of differential taxa. Red nodes represent taxa enriched in CTR group, blue nodes represent taxa enriched in ASD group, and yellow nodes represent taxa with no significant difference. **(C)** LEfSe LDA score bar plot showing biomarkers with statistical significance (LDA score ≥ 3.0,*P < 0.05). Orange bars represent taxa enriched in CTR group and blue bars represent taxa enriched in ASD group.

To identify specific species-level differences in gut microbiota between children with ASD and TD children, the relative abundances of species between the two groups were compared using the Wilcoxon rank-sum test. With a significance threshold of *P* < 0.05, a total of 15 differentially abundant species were identified, including 10 species significantly enriched in the CTR group and 5 species enriched in the ASD group (Figure 4A). These differential taxa represent key microbial contributors to gut microecological dysbiosis and the markedly reduced GMHI in ASD.

It should be noted that the characterization of these taxa as “health-associated” or “disease-associated” reflects population-level compositional patterns and established functional associations reported in prior literature, rather than definitive functional assignments in individual participants. Microbial functions are inherently strain-specific and context-dependent, and the roles of these taxa may vary according to host immune status, overall community composition, and local intestinal microenvironment. Species significantly enriched in the CTR group but depleted in the ASD group mainly included: (1) Members of *Bifidobacterium*:*Bifidobacterium bifidum* (*P* = 0.00113, ^**^) and *Bifidobacterium adolescentis* (*P* = 0.02304, ^*^). The significant depletion of *Bifidobacterium* suggests a loss of protective microbiota in the gut of children with ASD, which may impair intestinal anti-infective capacity and immune homeostasis. (2) Short-chain fatty acid (SCFA)-producing functional bacteria:*[Eubacterium] hallii* group (*P* = 0.00699, ^**^), uncultured bacterium from g__[Eubacterium] hallii group (*P* = 0.01228, ^*^), and *Anaerostipes hadrus* (*P* = 0.03343, ^*^). These taxa are important butyrate producers that generate butyrate via dietary fiber fermentation. As the primary energy source for colonic epithelial cells, butyrate exerts multiple biological effects, including maintaining intestinal barrier function, anti-inflammatory activity, regulating gene expression, and neuroprotection. (3) Other functional taxa: uncultured organism g_*Blautia* (*P* = 0.01802) belonging to Lachnospiraceae, which participates in complex carbohydrate degradation and SCFA production; uncultured organism g_Fusicatenibacter^*^ (*P* = 0.00165, ^**^), also a butyrate producer; metagenome g_*Rikenellaceae RC9 gut group* (*P* = 0.00198, ^**^), which contributes to intestinal metabolic homeostasis; *Streptococcus salivarius subsp. thermophilus* (*P* = 0.00143, ^**^), a probiotic strain that produces lactate and modulates the intestinal microenvironment; and metagenome g_Clostridium sensu stricto 1 (*P* = 0.00656, ^**^), involved in intestinal anaerobic fermentation (Figure 4A). The systematic reduction of these functional taxa reflects global defects in key gut microbiome functions in children with ASD, including carbohydrate metabolism, SCFA production, and intestinal homeostasis maintenance.

Species significantly enriched in the ASD group but relatively scarce in the CTR group mainly included: (1) Members of *Bacteroides*:*Bacteroides dorei* (*P* = 0.00341, ^*^), *Bacteroides vulgatus* (*P* = 0.01228, ^*^), and *Bacteroides uniformis* (*P* = 0.0011, ^**^) were all significantly elevated in the ASD group.As major Gram-negative anaerobic bacteria in the gut, *Bacteroides* participate in complex polysaccharide degradation and energy metabolism under physiological conditions. However, under dysbiotic conditions, overgrowth of certain Bacteroides strains has been associated with increased intestinal permeability and pro-inflammatory signaling in prior studies: their outer membranes are rich in lipopolysaccharide (LPS), and some strains produce neuroactive metabolites such as p-cresol, which has been associated with ASD symptom severity in previous investigations. It should be emphasized, however, that these effects are strain-specific and context-dependent, and cannot be generalized to all members of the Bacteroides genus. Thus, the concurrent enrichment of multiple Bacteroides species in the ASD group may, in the context of the overall dysbiotic community structure observed, be consistent with a pro-inflammatory microenvironmental tendency, though direct functional validation is required. (2) Members of *Parabacteroides*:*Parabacteroides merdae* showed significantly higher relative abundance in the ASD group than in the CTR group.Belonging to Bacteroidota together with *Bacteroides, Parabacteroides* shares similar metabolic traits. Its overabundance may be related to altered intestinal redox potential, modified mucus layer structure, or host immune selection pressure; its exact functional role in this study warrants further investigation. (3) Members of *Streptococcus*: uncultured organism g__*Streptococcus* was significantly increased in the ASD group.*Streptococcus* comprises various facultative anaerobes, some of which are opportunistic pathogens. Its enrichment may reflect shifts in the gut microenvironment (e.g., oxygen tension, pH, nutrient availability) in children with ASD, favoring facultative anaerobes rather than strictly anaerobic beneficial taxa (Figure 4A). Moreover, some streptococci can promote intestinal inflammation via hemolysins, superantigens, or inflammatory mediators, potentially worsening intestinal barrier dysfunction.

Building on the above analyses, linear discriminant analysis effect size (LEfSe) was further applied to systematically identify and evaluate differentially abundant taxa between ASD and CTR groups. The cutoff criteria were set at LDA score ≥ 3.0 and *P* < 0.05. In total, 16 differentially abundant taxa were identified. The phylogenetic cladogram (Figure 4B) illustrated their phylogenetic distribution, and the LDA score bar plot (Figure 4C) directly showed the effect size of each differential taxon: 10 taxa enriched in the CTR group and 6 in the ASD group. The functional characteristics of the enriched taxa were sharply contrasting: nearly all taxa enriched in the CTR group were known health-associated taxa, whereas those enriched in the ASD group were disease-associated taxa reported in the literature.

Most taxa enriched in the CTR group exerted well-defined beneficial functions and clustered into two core functional modules: (1) SCFA-producing bacteria, including *[Eubacterium] hallii*, uncultured organism g__*Blautia, Anaerostipes hadrus*, and metagenome g__*Clostridium sensu stricto 1*. Their coordinated enrichment indicates a robust and complete SCFA biosynthetic capacity in the healthy childhood gut, which is essential for barrier maintenance, energy supply, and immune regulation. (2) Classic probiotic strains, including *Bifidobacterium adolescentis* and *Streptococcus salivarius* subsp. *thermophilus*, which play key roles in pathogen exclusion, vitamin synthesis, and immune modulation. This consistent beneficial enrichment pattern represents a stable, functionally synergistic symbiotic microbiota in the healthy pediatric gut.

In contrast, most of the 6 taxa enriched in the ASD group are associated with disease states: (1) *Bacteroides uniformis*, which may exert pathogenic effects under certain conditions by inducing pro-inflammatory signaling via LPS or impairing the mucus barrier, potentially contributing to local and systemic low-grade inflammation in ASD. (2) Other disease-related taxa, including metagenome g__*Rikenellaceae RC9 gut group* and unclassified g__*Prevotella* 9, which have been reported to be enriched in patients with inflammatory or metabolic disorders. Their selective enrichment in the ASD group suggests that the dysbiotic gut environment in ASD may confer a growth advantage to these microbes. The enrichment of these opportunistic pathogens, combined with the depletion of health-associated taxa, likely exacerbates gut microbiota dysbiosis in children with ASD.

## 4 Discussion

This study revealed ASD-associated gut microecological dysbiosis across multiple dimensions including community structure, microbiome health indices, and differential taxa, using full-length 16S rRNA gene sequencing. Despite the absence of significant α-diversity differences, significant and consistent differences were identified in community composition, GMHI/MDI, and specific functional bacterial abundance, providing microbiological evidence relevant to gut-brain axis mechanisms in ASD.

At the phylum level, we observed a significantly reduced Bacillota/Bacteroidota ratio in the ASD group, consistent with the notion that an abnormal F/B ratio serves as a hallmark of microbial imbalance in various diseases (Zeidan et al., 2022; Ojima et al., 2016), and further supporting the presence of gut microecological dysbiosis in this cohort. In the present study, the decline in the relative proportion of Bacillota was primarily attributed to the depletion of SCFA-producing bacteria, whereas the increase in Bacteroidota abundance was driven by the overabundance of specific Bacteroides species. Such structural alterations at the phylum level not only reflect compositional changes in microbiota at the quantitative level, but also represent qualitative shifts in functional ecological niches.

These qualitative shifts in functional ecological niches are concretely manifested at the species level as follows. Most of the enriched taxa within Bacillota, such as members of Lachnospiraceae and Ruminococcaceae families, are major producers of intestinal SCFAs, and butyrate production in particular highly depends on these bacteria (Li et al., 2021). We found that multiple butyrate-producing bacteria were significantly depleted in the gut of children with ASD, including *[Eubacterium] hallii, Anaerostipes hadrus*, uncultured organism g__*Blautia*, and *Fusicatenibacter* spp. Butyrate can modulate central nervous system function through multiple pathways: partly crossing the blood-brain barrier to regulate neuronal activity (Ge et al., 2023), transmitting signals via the vagus nerve (Li et al., 2018), modulating hypothalamic-pituitary-adrenal (HPA) axis activity (van de Wouw et al., 2018), and influencing neurotransmitter synthesis and neurotrophic factor expression (Srivastav et al., 2019). Therefore, the systemic reduction of butyrate-producing bacteria may represent one of the key links connecting gut microbial dysbiosis with neurodevelopmental abnormalities in ASD. Meanwhile, pronounced compositional changes were also detected within Bacteroidota. Our study demonstrated significant enrichment of *Bacteroides dorei, Bacteroides vulgatus, Bacteroides uniformis*, and *Parabacteroides merdae* in the ASD group. Although *Bacteroides* spp. play an essential role in polysaccharide degradation in the healthy gut (Yu et al., 2025), excessive proliferation of specific strains may have adverse consequences. Furthermore, the restrictive dietary patterns frequently observed in children with ASD—present in 48.1% of our cohort—may independently reduce dietary fiber intake and subsequently alter gut microbiota composition, representing an important confounding factor that warrants consideration in interpreting these findings.

The absence of significant α-diversity differences alongside significant β-diversity, GMHI/MDI, and differential species findings highlights that species richness and evenness are relatively independent of species identity and function (Lin et al., 2025; Jo et al., 2022; Kadiyska et al., 2025). In ASD, dysbiosis appears to manifest primarily as a compositional redistribution—health-associated taxa replaced by disease-associated taxa—rather than global species loss. It should also be noted that while the β-diversity difference was statistically significant, the explained variance was modest (*R*^2^ = 0.01682), reflecting the multifactorial nature of gut microbiome variation and indicating that ASD group membership accounts for only a small proportion of total inter-individual microbiota variation. This underscores the need for larger sample sizes and comprehensive confounder control to achieve clinically meaningful effect sizes.

The significant GMHI and MDI differences (*P* < 0.001) demonstrate that integrating species identity with health or disease associations captures compositionally relevant information undetectable by α-diversity indices alone. The near-complete separation of GMHI values between groups reflects the consistency of ASD-associated compositional imbalance in this cohort. However, as GMHI was developed in adult multi-disease datasets, its application here is exploratory; independent validation in pediatric ASD cohorts is required before it can be considered a robust biomarker.

We identified a significant depletion of *Bifidobacterium bifidum* and *Bifidobacterium adolescentis* in the gut of children with ASD, a finding with important clinical translational implications. The genus *Bifidobacterium* is a key component of the early life gut microbiota, predominating during infancy and early childhood (Martin et al., 2016), and plays pivotal roles in immune system maturation (Laursen et al., 2021), metabolic development, and neural development (Du et al., 2025; Hunter et al., 2023). From an intervention perspective, the marked depletion of bifidobacteria provides a clear target for probiotic supplementation. Numerous clinical studies have explored the effects of probiotics on ameliorating ASD symptoms, with some reporting improvements in both gastrointestinal and behavioral symptoms (Shaaban et al., 2018; Guidetti et al., 2022). Nevertheless, the efficacy of probiotic interventions is heterogeneous, which may be attributed to strain specificity, dosage, treatment duration, and individual differences in gut microenvironment. Our study identified specific bifidobacterial species deficient in children with ASD *(B. bifidum* and *B. adolescentis*), providing a scientific basis for designing more targeted probiotic formulations. Future research should investigate whether supplementation with these specific strains can effectively colonize the gut of children with ASD, improve GMHI scores, and ultimately modify clinical phenotypes. However, it should be emphasized that improvement in GMHI scores following probiotic intervention has not yet been validated as a surrogate endpoint for clinical outcomes in ASD, and the relationship between GMHI changes and behavioral or neurodevelopmental improvement requires prospective investigation.

The observed compositional alterations may potentially contribute to gut-brain axis dysregulation through four pathways, though these represent evidence-informed hypotheses given the absence of metabolomic or biomarker data: (1) depletion of butyrate-producing bacteria reduces intestinal butyrate availability, impairing epithelial integrity and neuroprotective signaling; (2) dysbiosis-associated immune dysregulation may elevate pro-inflammatory mediators linked to neuroinflammation, pending direct immunological validation; (3) impaired intestinal barrier function may permit LPS translocation into systemic circulation, potentially compromising blood-brain barrier integrity; (4) microbial dysbiosis may alter the synthesis and availability of neuroactive compounds including GABA, serotonin, and their precursors, affecting neurodevelopment.

Notably, this study is a cross-sectional observational investigation and cannot establish causality between microbiota alterations and ASD. Possible scenarios include: (1) Microbial dysbiosis acts as an etiological factor of ASD, affecting neurodevelopment in early life; (2) Microbiota changes are secondary to genetic, immune, or metabolic abnormalities associated with ASD; (3) Microbial dysbiosis and ASD interact in a bidirectional positive feedback loop; (4) Both are influenced by common upstream factors such as genetic susceptibility and early-life environmental exposures. These limitations are reinforced by evidence questioning a direct pathogenic gut microbiota role in ASD: Yap et al. (*n* = 247) found that ASD-microbiome associations were largely mediated by dietary preferences rather than intrinsic dysbiosis (Yap et al., 2021); sibling-controlled meta-analyses demonstrated limited and unstable microbiota differences between ASD individuals and their neurotypical siblings (Xiao et al., 2025); and Korteniemi et al. identified dietary assessment, antibiotic exposure, and control group selection as major sources of heterogeneity across the literature (Korteniemi et al., 2023). Accordingly, the present findings should be regarded as hypothesis-generating observational evidence, and future longitudinal, sibling-controlled, multi-omics studies will be essential to establish the directionality of these associations.

This study has several notable strengths: a well-characterized cohort with matched baseline demographics, a multi-dimensional analytical framework integrating community structure, composite health indices, and differential taxa, and the use of full-length 16S rRNA sequencing on the PacBio Sequel IIe platform providing substantially improved species-level resolution compared with short-read approaches. Nevertheless, several limitations warrant consideration. First, the cross-sectional design precludes causal inference; longitudinal studies tracking microbiota trajectories relative to ASD development are needed. Second, full-length 16S rRNA sequencing does not afford strain-level functional genomic information; functional inferences are based on taxonomic composition and PICRUSt2 predictions and require validation through shotgun metagenomics and metabolomics. Third, quantitative dietary data were not collected and gastrointestinal symptoms were not incorporated as covariates, limiting the ability to disentangle ASD-specific from diet- or GI-driven microbiota differences. Fourth, the sample size of 100, while comparable to existing ASD microbiome studies, limits power for subgroup analyses by severity, age, or comorbidity. Fifth, the single-center eastern China design limits generalizability; multicenter replication is necessary. Sixth, GMHI and MDI were developed in adult multi-disease cohorts and require independent validation in pediatric ASD populations. Finally, the absence of functional validation means findings remain predominantly descriptive.

Future research should conduct multi-omics integration studies combining metagenomics, metabolomics, transcriptomics, and immunomics to construct a multi-level regulatory network of the gut-brain axis. Additionally, germ-free animal models and fecal microbiota transplantation experiments should be utilized to verify the causal effects of specific microbiota or metabolites on ASD-related behaviors and neurobiological markers. Furthermore, personalized probiotic/prebiotic/dietary intervention regimens based on individual microbiota signatures should be designed to evaluate their impacts on gut health, GMHI values, and clinical symptoms. Associations between microbiota profiles and ASD subtypes, symptom severity, and comorbidity patterns (e.g., gastrointestinal symptoms) should also be explored to achieve microbiota-based precise stratification of ASD.

## 5 Conclusions

Using full-length 16S rRNA gene sequencing on the PacBio Sequel IIe platform, this study systematically compared the gut microbiota characteristics between children with ASD and typically developing (TD) children, and identified an ASD-associated pattern of gut microbial dysbiosis. No significant differences were observed in α-diversity indices between groups; however, marked differences were detected in β-diversity, community composition, and microbial health status. At the phylum level, the Bacillota/Bacteroidota ratio was significantly decreased in the ASD group, indicating substantial alterations in gut microbial structure. GMHI and MDI analysis revealed that the overall gut microbiome health status was significantly lower in the ASD group, with near-complete separation between the two cohorts. Differential species analysis identified 15 significantly different taxa, demonstrating a bidirectional compositional imbalance characterized by systematic depletion of health-associated functional taxa and selective enrichment of taxa with reported associations with dysbiotic or inflammatory states—though such characterizations reflect population-level patterns from prior literature and are subject to strain- and context-dependent variation. LEfSe analysis further validated 16 significantly differential taxa: microbes enriched in the TD group were mainly SCFA producers and classic probiotic species, whereas those enriched in the ASD group were predominantly taxa with reported associations with inflammatory or metabolic conditions.

These findings provide microbiological evidence relevant to understanding gut-brain axis mechanisms in ASD and offer a preliminary observational basis for hypothesis-driven investigation of microbiota-targeted strategies. The significant depletion of Bifidobacterium spp. and butyrate-producing bacteria provides a scientific rationale for exploring targeted probiotic and prebiotic interventions, though the efficacy and clinical relevance of such strategies require prospective validation. It should be noted that the cross-sectional design of this study precludes causal inference; whether the observed microbiota differences represent causes, consequences, or correlates of ASD cannot be determined from the present data, and the possibility that these differences are partly attributable to ASD-related dietary restriction or gastrointestinal comorbidities cannot be excluded. Future longitudinal studies, multi-omics integration, mechanistic validation using animal models, and rigorously controlled clinical intervention trials are warranted to clarify the directionality of gut microbiota alterations in ASD and to evaluate the potential of microbiota-based approaches as adjuvant strategies.

## Data Availability

The raw 16S rRNA amplicon sequencing data generated during this study have been deposited in the NCBI Sequence Read Archive (SRA) under BioProject accession number PRJNA1503725. Additional data supporting the conclusions of this article will be made available by the corresponding author, without undue reservation.
