# Correction: A case-control analysis: changes in gut microbiota composition in children with autism spectrum disorder

**DOI:** 10.3389/fmicb.2026.2006584

**Published:** 2026-10-13

**Authors:** Linlin Fan, Ying Tian, Jiayi Jin, Na Zhang, Caihong Sun, Yang Liu, Le Kang

**Affiliations:** 1 Department of Child Health Care, Yantai Affiliated Hospital of Shandong Medical and Pharmaceutical University, Yantai, China; 2 Department of Neonatology, Yantai Affiliated Hospital of Shandong Medical and Pharmaceutical University, Yantai, China; 3 School of Pharmacy, Shandong Medical and Pharmaceutical University, Yantai, China; 4 Department of Gynecology, Jiaoqiao Central Health Center, Jiaoqiao, China; 5 School of Special Education and Rehabilitation, Shandong Medical and Pharmaceutical University, Yantai, China; 6 Department of Hand Surgery & Microsurgery and Foot & Ankle Surgery, Yantai Affiliated Hospital of Shandong Medical and Pharmaceutical University, Yantai, China

**Keywords:** 16S rRNA sequencing, autism spectrum disorder, case-control study, gut microbiota, microbial dysbiosis

In the published article, the authors were listed in the wrong order.

The correct author list is as follows: “Linlin Fan, Ying Tian, Jiayi Jin, Na Zhang, Caihong Sun, Yang Liu and Le Kang.”

The original article has been updated.

